# Prevalence of comorbidity of chronic diseases in Australia

**DOI:** 10.1186/1471-2458-8-221

**Published:** 2008-06-27

**Authors:** Gillian E Caughey, Agnes I Vitry, Andrew L Gilbert, Elizabeth E Roughead

**Affiliations:** 1 Quality Use of Medicines and Pharmacy Research Centre (QUMPRC), Sansom Institute, School of Pharmacy and Medical Sciences, University of South Australia, Adelaide, Australia

## Abstract

**Background:**

The prevalence of comorbidity is high, with 80% of the elderly population having three or more chronic conditions. Comorbidity is associated with a decline in many health outcomes and increases in mortality and use of health care resources. The aim of this study was to identify, review and summarise studies reporting the prevalence of comorbidity of chronic diseases in Australia.

**Methods:**

A systematic review of Australian studies (1996 – May 2007) was conducted. The review focused specifically on the chronic diseases included as national health priorities; arthritis, asthma, cancer, cardiovascular disease (CVD), diabetes mellitus and mental health problems.

**Results:**

A total of twenty five studies met our inclusion criteria. Over half of the elderly patients with arthritis also had hypertension, 20% had CVD, 14% diabetes and 12% mental health problem. Over 60% of patients with asthma reported arthritis as a comorbidity, 20% also had CVD and 16% diabetes. Of those with CVD, 60% also had arthritis, 20% diabetes and 10% had asthma or mental health problems.

**Conclusion:**

There are comparatively few Australian studies that focused on comorbidity associated with chronic disease. However, they do show high prevalence of comorbidity across national health priority areas. This suggests integration and co-ordination of the national health priority areas is critical. A greater awareness of the importance of managing a patients' overall health status within the context of comorbidity is needed together with, increased research on comorbidity to provide an appropriate scientific basis on which to build evidence based care guidelines for these multimorbid patients.

## Background

The proportion of Australians aged 65 years and over is projected to increase from 2.6 million in 2004, to over 6.5 million by 2051, with the highest projected growth rate for those aged 80 years or older [1]. This ageing Australian population, with a concomitant rise in the number of people living with chronic diseases, has major implications for both health care services and their associated costs. Chronic diseases are the leading cause of illness and disability in those aged 65 years and over [2] and in 2000–01 accounted for nearly 70% of all health system expenditure in Australia (over $AU 35 billion) [3-5]. The World Health Organisation estimates that chronic disease accounts for 60% of deaths worldwide and has given precedence globally to the prevention and treatment of chronic disease [6].

The co-existence of multiple chronic diseases is common especially in the older population [7]. The 2004–2005 Australian National Health Survey (NHS) reported almost all Australians aged 65 years or older have at least one chronic condition, with 80% reported as having three or more chronic conditions [2,5]. Similar burdens have been reported in other countries, including the USA and Canada [8,9]. The presence of multiple chronic diseases is associated with a decline in many health outcomes, including quality of life, mobility, functional ability, and increases in hospitalisations, psychological distress, mortality and the use of health care resources [8,10-12].

The study of multimorbidity that is, the presence of two or more chronic diseases or conditions within one person is relatively new worldwide [13] and a recent editorial highlights the importance and growing awareness of this endemic health problem, particularly with regard to focusing worldwide research efforts in this area [14]. When multimorbidity is studied in relation to a particular disease or 'index' disease, the term co-morbidity should be used [15,16].

As a result of multimorbidity, polypharmacy in the elderly is common. In Australia, almost 88% of those aged 65 years and over use at least one prescription medication [17] with reports of between 43–55% of elderly patients taking 4 or more medications regularly [17,18]. With increasing numbers of concurrent medications, the risk of adverse drug events increases significantly. It has been estimated that the likelihood of an older person having an adverse drug event increases from 10% if one medication is taken, to 75% if five or more medications are used [18]. The incidence of medication-related problems and adverse outcomes is increasing, especially in the elderly [19-21].

While clinical guidelines have been developed to help health professionals with the choice of appropriate treatments, their relevance to the care of those with multiple chronic diseases, particularly the elderly, has recently been questioned [22-24]. Current evidence-based guidelines often recommend several medications in the treatment of individual chronic diseases, resulting in potentially very complex regimens in those with multiple morbidities. Moreover the evidence is largely based on relatively short-term randomised clinical trials of single conditions and often exclude the elderly or those with multimorbidity [25]. A detailed understanding of the prevalence of multimorbidity and the common comorbidities associated with chronic diseases in Australia may help to highlight the complex issues surrounding the management of these patients.

The aim of this study was to identify, review and summarise studies describing the prevalence of multimorbidity and comorbidity of chronic diseases in Australia over the last decade. With a focus specifically on the chronic diseases defined as Australian National Health Priority Areas (NHPA), which include arthritis, asthma, cancer, cardiovascular disease (CVD), diabetes, and mental health problems (particularly depression) [26]. These NHPA account for at least 60% of the total burden of disease and injury and approximately $20 billion or 42% of allocated health system expenditure in Australia [27].

## Methods

### Search Strategy

A systematic search of Medline (via Ovid) was conducted for eligible articles published between January 1996 – May 2007, inclusive. Searches were performed using MeSH subject headings where relevant. The primary search terms included "multimorbidity", "comorbidity", "polymorbidity", "chronic condition", "chronic disease" coupled with "Australia" or each of the states and capital cities, to limit the search to work undertaken in Australia. The search strategy also included the six chronic diseases of interest "arthritis", "asthma" (and "chronic obstructive pulmonary disorder" (COPD)), "cancer", "cardiovascular diseases" (including ischemic heart disease, coronary heart disease, hypertension, heart failure, coronary syndrome, cerebrovascular disease), "diabetes mellitus", and "mental disorders" (including depression, affective disorders, anxiety) together with the primary search terms or in combination with one another and limited to Australian studies.

In addition, websites of The Australian Institute of Health and Welfare (AIHW), Commonwealth and all state government departments (e.g. The Department of Health) and the Australian Bureau of Statistics (ABS) were searched to locate relevant publication and reports. Reference lists of included studies were also searched to identify additional sources of information.

### Inclusion/Exclusion Criteria

Only Australian studies where the prevalence of multimorbidity or comorbidities associated with the chronic conditions detailed above were reported were included. Articles were excluded if the search terms were only mentioned once in the introduction or discussion of the article. The recent National Health Survey (NHS) 2004–05 was included and results from previous NHS were excluded. The NHPA of mental health, focuses specifically on depression, and the broad classification of affective disorders were included in this study. Substance abuse disorders were excluded. Studies that reported depression or anxiety at the time of hospitalisation for an acute CVD episode were excluded. Studies scoring < 4 using the critical appraisal tool (see below) were deemed to be of low quality and as such were excluded from the review.

### Methodological Appraisal

An assessment of methodological rigour was conducted using the critical appraisal guidelines for research articles reporting prevalence, developed by Loney et al [28]. The scoring system consisted of 8 dichotomous questions, with 1 for yes and 0 for no, on the validity of the study design (appropriate sampling methods and frame, adequate sample size, appropriate outcomes measurements and response rates), interpretation (prevalence given with confidence intervals, adequate subgroup analysis) and applicability of the results (study subjects and setting described in detail). All questions were weighted equally with higher scores reflecting better methodological quality of the studies.

## Results

### Study Characteristics

**Figure 1:**
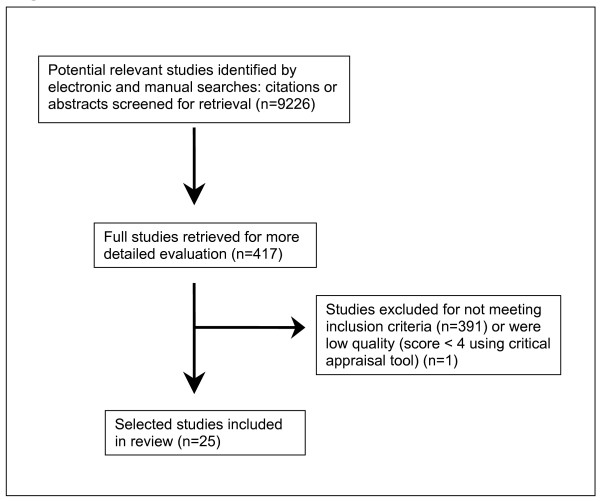
Summary of search results.

A summary of the results of the search is provided in Figure 1, which outlines the number of potentially relevant studies initially identified and the loss to inclusion criteria. Overall, a total of twenty five studies met our inclusion criteria across the six chronic diseases of interest. There were no Australian studies that reported the prevalence of multimorbidity. All of the 25 eligible studies reported on the prevalence of co-morbidities of people with an index chronic disease. The results were therefore divided into each index chronic disease and then further grouped according to the age of the studies participants. These age groupings were divided into those studies that looked specifically at elderly (≥ 65 years old, and the general adult population, where applicable). In two instances data from one study included both the elderly and the adult populations [2,29].

The main findings from these studies including the study population, type of data collected, prevalence of both the index disease and co-morbidities and the critical appraisal scores are summarised in Tables 1, 2, 3, 4, 5, 6. The median quality score of the 25 studies included in this review was 5 points out of a maximum score of 8 (range 4–7 points). The main areas of methodological shortcomings of the reviewed studies included inadequate response rates and descriptions of the non-responders (61.5% of the studies) followed by 46%–58% of studies reporting inappropriate sampling methods and biased sample frames, respectively.

**Table 1:** Summary of studies reporting comorbidities with arthritis.

**Comorbidity Study**	Study Quality Score/8	Asthma (%)	CVD (%)	Diabetes (%)	Dyslipidemia (%)	Hypertension (%)	Malignancy (%)	Mental problem/ depression^#^ (%)	Osteoporosis (%)
Arthritis									

Hill et al, 2006 [29] ≥ 55 yrs n = 2,054 self-report of doctor diagnosed arthritis (prevalence 43.9%) and mental health condition	6	-	-	-	-	-	-	13.1	-
									
NHS 2004–05 [2] ≥ 65 yrs n = 3,212 self-report of all conditions (prevalence arthritis 49.4%)	7	12.1	21.5	14.1	-	-	6.9	8.8	-
									
O'Halloran et al, 2003 [42] ≥ 65 yrs n = 2,976 GP encounters (OA^a ^prevalence 20.9%)	5	-	18.7 (IHD^a^)	13.9	18.7	50.8	-	12.9^#^	11.5

Hill et al, 2006 [29] ≥ 18 yrs n = 7,473 self-report of doctor diagnosed arthritis (prevalence 18.3%) and mental health condition	6	-	-	-	-	-	-	14.9	-
									
NHS 2004–05 [2] ≥ 25 yrs n = 17,202 self-report of all conditions (prevalence of arthritis 22.8%)	7	13.2	12.7^b^	9.3^b^	-	-	5.0	13.3	-
									
Waghorn et al, 2006 [35] 15–64 yrs n = 37,580 self-report 1998 SDAC^c ^(prevalence arthritis 9.2%)	5	-	-	-	-	-	-	6.0d	-

-, not reported; #, depression only;^a ^OA, osteoarthritis; IHD, ischaemic heart disease; ^b ^≥ 45 years, data for 25 years and over were reported to have a high standard error and were deemed to be used with caution^2^; ^c ^SDAC; ABS Survey of Disability, Ageing and Carers; ^d ^depression and anxiety.

**Table 2:** Summary of studies reporting comorbidities with asthma.

**Comorbidity Study**	Study Quality Score/8	Arthritis (%)	CVD (%)	Diabetes (%)	Hypertension (%)	Malignancy (%)	Mental problem/ depression^#^ (%)	Osteoporosis (%)
Asthma								

Adams et al, 2006 [30] ≥ 55 yrs n = 2050 self-report of doctor diagnosed asthma and comorbidities (asthma prevalence 9.6%)	6	56.9	26.9 (HD)^a^ 9.1 (Stroke)^b^	12.1	-	17.7	-	17.3
								
NHS 2004–05 [2] ≥ 65 yrs n = 3,212 self-report all conditions (asthma prevalence 9.41%)	7	63.4	20.1	16.0	-	5.7	8.1	-

Adams et al, 2006 [30] ≥ 18 yrs n = 7,443 self-report of doctor diagnosed asthma and comorbidities (asthma prevalence 11.2%)	6	21.4.	6.9 (HD)^a^ 1.5 (Stroke)^b^	5.3	-	5.5	-	4.7
								
Goldney et al, 2003 [31] ≥ 15 yrs (mean age 44 yrs) n = 3010 self-report of doctor diagnosed asthma (prevalence 9.9%), depression assessed by PRIME-MD questionnaire^c^	6	-	-	-	-	-	22.1# (all depression) 14.4# (major depression)	-
								
NHS 2004–05 [2] ≥ 25 yrs n = 17,202 self-report all conditions (asthma prevalence 9.40%)	7	32.0	6.7^d^	6.1^d^	-	3.2^d^	15.0	-
								
Pilotto et al, 2004 [36] ≥ 18 yrs (mean age 48 yrs) n = 170 GP recruitment of asthma patients, self-report of comorbidities	4	-	11.8^e^	7.1	18.2	-	-	6.5

-, not reported; #, depression only; ^a^HD, Heart Disease; ^b^Stroke; ^c^PRIME-MD, primary care evaluation of mental disorders questionnaire, a validated tool used to diagnose depression/mental health disorders; ^d ^≥ 45 years, data for 25 years and over were reported to have a high standard error and were deemed to be used with caution^2^; ^e^angina, heart failure or other heart disease.

**Table 3:** Summary of studies reporting comorbidities with cancer.

**Comorbidity Study**	Study Quality Score/8	Arthritis (%)	Asthma (%)	CVD (%)	Diabetes (%)	Hypertension (%)	Mental health problem (%)
Cancer							

NHS 2004–05 [2] ≥ 65 yrs n = 3,212 self-report all conditions (malignant neoplasm prevalence 6.1%)	7	55.7	15.1	14.6	9.0	-	16.1

NHS 2004–05 [2] ≥ 25 yrs n = 17,202 self-report all conditions (malignant neoplasm prevalence 1.7%)	7	43.8	6.1	18.3	8.8	-	7.1
							
Valery et al, 2006 [52] all ages (85% ≥ 40 yrs) n = 810, non-indigenous patients identified from cancer registry, comorbidities hospital administrative data and medical records	5	-	12.0 (Respiratory Disease)	5.0 (ACS)^a^	6.0	8.0	-

-, not reported; ^a ^ACS; Acute Coronary Syndrome

**Table 4:** Summary of studies reporting comorbidities with diabetes.

**Comorbidity Study**	Study Quality Score/8	Arthritis (%)	Asthma (%)	CVD (%)	Dyslipidemia (%)	Hypertension (%)	Malignancy (%)	Mental problem/ depression^#^ (%)	Osteoporosis (%)
Diabetes									

O'Halloran et al, 2003 [42] ≥ 65 yrs n = 2,976 GP encounters (diabetes prevalence 14.4%)	5	20.1 (OA)^a^	-	23.6 (IHD)^a^	22.2	52.7	-	9.0^#^	13.8
									
NHS 2004–05 [2] ≥ 65 yrs n = 3,212 self-report all conditions (diabetes prevalence 13.7%)	7	51.0	11.0	26.7	-	-	5.4	7.2	
									
Thomas and Nestel, 2007 [43] mean age = 65 yrs, n = 3893 NEFRON study, GP diagnosed type 2 diabetes and comorbidities	4	-	-	40.1^b^	-	-	-	-	-

Bruce et al, 2005 ≥ 40 yrs [38] (mean age 64 yrs) n = 1,273 clinically diagnosed type 2 diabetes, depression GHS^c ^questionnaire, comorbidities self-report validated with hospital records	5	-	-	41.5^d^	-	51.1	-	31.5^#^	
									
Clarke et al, 2006 [40] ≥ 35 yrs (mean age 64 yrs), n = 20,538, diabetes diagnosed by diabetes medication, HbA1c or hospital diagnosis (primary or secondary ICD-9 250), co-morbidities identified from hospital administrative data (ICD-9 CM)	6	4.0^a^ (Musc. Dis)	2.9^a^ (Resp Dis)	12.3^e^	-	-	5.6	0.9	
									
Goldney et al, 2004 [32] ≥ 15 yrs n = 3,010 self-report of doctor diagnosed diabetes (prevalence 5.2%), depression by PRIME-MD^f ^questionnaire.	5							23.6^#^	
									
Kemp et al, 2005 [33] ≥ 25 yrs, n = 11,247 AusDiab survey, self-report doctor diagnosed diabetes, confirmed with hypoglycemic drug use or glucose tolerance test (diabetes prevalence 3.9%), clinical measurement of comorbidities.	5	-	-	-	47.1	53.8	-	-	-
									
Mitchell et al, 1998 [34] ≥ 49 yrs, n = 3,654 self report of doctor diagnosed diabetes (prevalence 5.9%) and comorbidities	6	-	-	54.7	-	57.6	-	-	-
									
NHS 2004–05 [2] ≥ 25 yrs n = 17,202 self-report all conditions (diabetes prevalence 5.2%)	7	45.4^g^	12.3^g^	20.9	-	-	4.8	9.0	

-, not reported; #, depression; ^a ^(OA, osteoarthritis; IHD, ischemic heart disease; CHD, coronary heart disease; CV, cervebrovascular disease; PVD, peripheral vascular disease; Musc. Dis, Musculoskeletal disease (ICD 710–739); Resp. Dis, Respiratory Disorder (ICD 460–519));^b ^(coronary heart disease, cerebrovascular disease and peripheral vascular disease); ^c ^General Health Status (GHS) questionnaire, those reported as having at least 2 depressive symptoms were classed as having depression; ^d ^coronary heart disease and cerebrovascular disease; ^e ^angina, AMI and stroke; ^f ^PRIME-MD; primary care evaluation of mental disorders questionnaire, a validated tool used to diagnoses depression/mental disorders; ^g ^≥ 45 years, data for 25 years and over were reported to have a high standard error and were deemed to be used with caution^2^.

**Table 5:** Summary of studies reporting comorbidities with cardiovascular disease.

**Comorbidity Study**	Study Quality Score/8	Arthritis (%)	Asthma (%)	CVD (%)	Diabetes (%)	Dyslipidemia (%)	Hypertension (%)	Malignancy (%)	Mental problem/ depression^#^ (%)
Cardiovascular Disease									

NHS 2004–05 [2] ≥ 65 yrs n = 3,212 HSVD^a ^self-report all conditions (prevalence 18.4%)	7	57.9	10.3	•	19.8	-	-	8.4	10.5
Krum et al, 2006 [44] mean age 64 yrs, n = 479, admission to hospital for MI^a^, comorbidities from hospital administrative data and records	4	-	-	24.2 (CHF^a^)	21.6	52.9	53.4	-	-
O'Halloran et al, 2003 [42] ≥ 65 yrs n = 2,976 GP encounters (IHD^a ^prevalence 16.9%)	5	23.1 (OA)	-		20.1	23.1	47.3	-	9.5^#^
Parker et al, 2006 [37] mean age 63 yrs, n = 489 hospital admission for ACS^a^, depression assessed by CIDI^b^, comorbidities self-report	5			34.6 (pMI^a^)	17.3		63.5		10.6
Simons et al, 2002 [45] ≥ 60 yrs n = 2,217 (Dubbo study) hospital admission for MI^a ^(prevalence 18.7%), comorbidities from medical history and clinical examination	6	-	-	36.0 (pCS^a^)	16.0	26.9	62.0	-	
Krum et al, 2001 [46], ≥ 60 yrs, n = 22,060, GP diagnosis CHF^a ^(prevalence 13.2%), comorbidities from hospital administrative data and records	5	-	-	44.8 (angina) 29.7 (pMI^a^)	-	-	68.3	-	-
Stewart et al, 2001 [41] ≥ 55 yrs (mean age 76 yrs) n = 200 hospital admission CHF^a^, comorbidities hospital discharge data	4	-	36.0 (CAD^a^)	78.0 (IHD^a^) 70.0 (pMI)	32.0	-	66.0	-	-
O'Halloran et al, 2003 [42] ≥ 65 yrs n = 2976 GP encounters (HT^c ^prevalence 45.6%)	5	23.3 (OA)	-	17.5 (IHD^a^)	16.7	23.7	•	-	9.0#
Sturm et al, 2004 [47] mean age 71 yrs, n = 226 (NEMESIS)^a^, stroke detected & diagnosed by medical practitioner, comorbidities by past medical history and/or current presentation (diabetes or AF^a^)	5	-	-	23.5 (AF^a^) 6.2 (PVD^a^) 8.8 (pMI^a^)	19.1	-	58.9	-	-

NHS 2004–05 [2] ≥ 25 yrs n = 17,202 HSVD self-report all conditions (prevalence 5.7%)	7	54.4^d^	12.6	•	19.2	-	-	7.0^d^	14.0^d^
Powell et al, 2001 [48], 20–85 yrs (mean age 66 yrs) n = 1765, hospital admission for HD (AMI, angina, IHD, CHF)^c ^ICD-9 CM, comorbidities extracted from medical records and coded to corresponding ICD-9 CM diagnosis codes	4	2.2 (RA^a^)	14.6 (CAL^a^)	12.7 (Arr^a^) 11.5 (CV^a^) 8.2 (PVD^a^)	18.4	22.8	47.7	3.3	-
Burvill et al, 1997 [39] all ages, n = 191 (Perth community stroke study), first ever stroke (physician diagnosis), self-report co-morbidities at time of stroke^e^	4	-	-	18.0 (pMI) 31.0 ((angina)	12.0	-	62.0	-	8.2^#*e*^

-, not reported; ^a^(HSVD, Heart, Stroke and Vascular Disease, includes ischaemic heart disease (IHD), cerebrovascular disease (CV), oedema and chronic heart failure (CHF) and diseases of the arteries, arterioles and capillaries), MI myocardial infarction, ACS acute coronary syndrome (includes MI or unstable angina), pMI previous MI, pCS prior coronary syndrome, CAD chronic airways disease, NEMESIS North East Melbourne Stroke Incidence Study, AF atrial fibrillation, PVD peripheral vascular disease, HD heart disease, RA rheumatoid arthritis, CAL chronic airway limitation (asthma, emphysema), Arr arrhythmia; ^b ^Current depression assessed by Composite International Diagnostic Interview (CIDI) used the DSM symptom and duration criteria that included at least two or more weeks of major depression and/or two or more years of dysthmia, hence excluded individuals that developed depression at or around the time of their ACS and admission; ^c ^HT; hypertension defined as systolic blood pressure ≥ 140 mmHg or diastolic blood pressure ≥ 90 mmHg, or use of antihypertensive drugs; ^d ^≥ 45 years, data for 25 years and over were reported to have a high standard error and were deemed to be used with caution^2^; ^e ^n = 208, for study cohort that includes those depressed (n = 17) at time of stroke.

**Table 6:** Summary of studies reporting comorbidities with mental health problem/depression.

**Comorbidity Study**	Study Quality Score/8	Arthritis (%)	Asthma (%)	CVD (%)	Diabetes (%)	Hypertension (%)	Dyslipidemia (%)	Malignancy (%)	Mental problem/ depression (%)
Mental health problem/depression									

O'Halloran et al, 2003 [42] ≥ 65 yrs n = 2,976, GP encounters (depression prevalence 10%)	5	27.0 (OA)	-	15.9 (IHD)	12.9	40.9	18.9	23.7	•
NHS 2004–05 [2] ≥ 65 yrs n = 3,212 mental health and behavioural problems^a^, self-report all conditions (prevalence 9.5%)	7	30.0	5.28	26.7	6.8	-	-	6.3	•

Hunt et al 2004 [49] ≥ 18 yrs n = 10,241, generalised anxiety disorder diagnosed by composite international diagnostic interview (CIDI) (prevalence 4.5%) depression CIDI	4	-	-	-	-	-	-	-	38.9 (depression)
NHS 2004–05 [2] ≥ 25 yrs n = 17,202 mental health and behavioural problems^a^, self-report all conditions (prevalence 12.1%)	7	30.3	14.1	14.0	4.7	-	-	6.3	•
Wilson et al 2003 [50] ≥ 15 yrs n = 382 GP diagnosed depression (Medic-GP database), comorbidities from medical records	5	-	-	-	-	-	-	-	8.9 (anxiety)
National Survey of Mental Health and Wellbeing, 1997 [51] ≥ 18 yrs n = 10,641 mental health problem diagnosed by CIDI (prevalence 12.3%)	7								
Affective disorder (prevalence 3.8%)		-	-	-	-	-	-	-	47.3 (anxiety)
Anxiety disorder (prevalence 5.5%)		-	-	-	-	-	-	-	24.5 (affective)

-, not reported; ^a^Mental and behavioural problems include alcohol and drug, affective, anxiety-related, psychological development, behavioural and emotional, organic mental problems and symptoms and signs involving cognition, perception, emotional state and behaviour.

There were three types of data commonly used and included self report, medical records and administrative date, either exclusively or in combination. Eleven studies used self-report of chronic conditions, either based on a diagnosis by a medical practitioner [2,29-34] or general subject self-report [2,35-39]. The NHS 2004–05 data was based on self-report of medically diagnosed conditions, except for mental health problems, that were participant self-report and therefore may be based on self-diagnosis [2]. Only two studies validated the self-report [33,38].

Twenty studies used medical records/clinical data. Medical records were used to identify patients diagnosed with chronic disease either for the index disease itself [36,39-41] and/or the co-morbidities [31-33,37,38,42-52]. Validated diagnostic questionnaires were used to diagnose mental health disorders, specifically depression [31,32,37,38,49,51] or anxiety [49,51].

Five studies used administrative records/data that were based on hospital separations [40,41,44,46,52].

### Co-morbidities of Chronic Disease

#### Arthritis

Four studies examined comorbidities in patients with arthritis [2,29,35,42] (Table 1). Over half of the elderly patients with arthritis had hypertension, followed by CVD (20%), dyslipidemia (18.7%), diabetes (14%) and mental health problem/depression (12%).

#### Asthma

The 2004–5 NHS reported over 60% of elderly patients with asthma reported arthritis as a comorbidity followed by CVD (20.1%) and diabetes (16%) (Table 2) [2]. In three studies of all adults with asthma, almost one third of patients had arthritis [2,31,36]. Mental health was also common, its prevalence ranged from 14–22% when determined from self-report [2] or by questionnaire [31]. Hypertension affected almost 20% of patients, followed by CVD (12%) in a study of GP recruited asthma patients [36]. There were no studies that examined the prevalence of comorbidity associated with COPD nor all respiratory diseases.

#### Cancer

Over half of the patients with cancer from the 2004–5 NHS reported arthritis as a comorbidity followed by mental health problems (16.1%), asthma (15.1%) and CVD (14.6%) (Table 3)[2]. Arthritis was the most common comorbidity of cancer patients across all ages (43.8%), followed CVD (18.3%) [2] and the broad classification of respiratory diseases (12%) [52].

#### Diabetes

In accordance with the documented association of diabetes with CVD and its associated risk factors including hypertension and dyslipidemia [53], these were prominent co-morbidities of diabetic elderly patients (Table 4) [2,42,43].

Arthritis was present in over half of those with diabetes from the 2004–05 NHS [2], and in 20% of diabetic patients presenting to their GP [42] and depression was present in approximately 10% of elderly patients with diabetes [42].

When extended to the adult population with diabetes, similar associations of CVD, hypertension and dyslipidemia as common comorbidities were observed [2,32-34,38,40]. Hypertension was the most prevalent comorbidity (51–58%) whether determined from clinical examination, self-report or hypertension medications [33,34,38]. Prevalence rates of CVD were approximately 40–55% in two studies of self-report [34,38] but only 12% from hospital administrative data [40] in diabetic patients. Arthritis was reported by almost half of the diabetics [2] and the prevalence of depression was 24%–32% [32,38].

#### Cardiovascular Disease

Cardiovascular disease (CVD) encompasses a wide range of disorders and Table 5 is further sub-divided into all CVD; acute myocardial infarction (AMI) or acute coronary syndromes (ACS) or ischaemic heart disease (IHD); chronic heart failure (CHF); hypertension (HT); and stroke. The reporting of comorbidities were similar, irrespective of the type of data used and age of the subjects (Table 5). HT was a key comorbidity for half those with CVD. Specific CVD states different from the 'index' CVD condition were common co-morbidities. For example in patients with MI, CHF was a co-existent CVD in almost a quarter [44]. Dyslipidemia and diabetes were also prominent comorbidities, present in 20% of those with CVD and approximately 20 – 55% of patients with CVD had arthritis [2,37,41,42,44-47].

#### Mental health problems

CVD, hypertension and dyslipidemia affected approximately 25%, 40% and 20% of older patients, respectively with mental health problems. Arthritis was also reported by 30% of patients [2,42]. When expanded to the adult population, depression and anxiety were prevalent comorbidities, as determined by diagnostic interviews for mental health [49,51]. From the 2004–05 NHS, arthritis was the most common comorbidity (30%), followed by asthma (14.1%) and CVD (14%) in subjects with mental health problems.

## Discussion

The major impact of chronic diseases, with regard to their large disease burden and associated costs, has placed them at the fore of international and Australian strategic health priorities [6,26]. Integral to this is the increasing number of patients with multimorbidity of chronic conditions. Despite the high prevalence of multimorbidity, particularly in the elderly, there are comparatively few Australian studies that focus on multimorbidity or comorbidities associated with chronic disease. What this review shows, however, is that there is much cross over of diseases that are considered national health priority diseases for Australia. For more than one in ten people and often more than one in five, arthritis is found to be co-existent with either CVD, diabetes, and mental health problems. Asthma is also commonly co-existent with heart disease and diabetes. This suggests integration and co-ordination of Australia's national health priority areas will be critical for improving health within each priority area.

Primary care physicians are the major providers of care for patients with multimorbidity [54]. Arthritis, HT, CVD and diabetes account for over 55% of chronic conditions managed by GPs in Australia for the elderly [42]. Based on the results from this review, potentially for every elderly patient presenting to their GP with a chronic disease, one in two will also have arthritis or HT, and one in five will have a type of CVD or diabetes. When placed in this context, the need for a greater awareness from both the physician and patient of the importance of managing a patients' overall health status within the context of multiple disease states, rather than a single disease entity, is highlighted. The majority of research on chronic disease is based on single index disease states, which is clearly inappropriate for patients with multimorbidity. The number of evidence based studies in treating patients with multiple chronic diseases, severely limits the ability to translate research findings into clinical practice for this population.

Patients with multimorbidity need coordinated and continuing care that takes into account patient preferences and disease severity. Patients with chronic disease make daily decisions about the management of their diseases [55] and patient self-management is essential for effective chronic illness care and improved patient outcomes [55,56]. There is increasing evidence that patients, in particular the elderly, vary in the emphasis they place on certain aspects of their health care, including mortality, quality of life, risk of adverse events and medication adherence [57]. From a patient's viewpoint, the effective management of symptomatic conditions may take precedence over asymptomatic conditions, despite the fact that treatment of this asymptomatic condition may result in decreased mortality with time. For example, symptomatic treatment of osteoarthritis with an NSAID may result in the greatest impact on the patient's quality of life but should it take priority over the management of their HT? There is a clear need for integrated care for patients with multimorbidity.

The benefits of disease-specific guidelines for the prescribing of medications are well recognised. However, the inclusion of strategies to treat or even account for multimorbidity in evidence-based disease specific guidelines is rarely evident [22-24], and provide physicians little guidance about caring for such patients.

The NHS (2004–05) was an important source of data in this review, based largely on self-report of a health professional diagnosis. However, the majority of studies that met our inclusion criteria were from clinical research studies that concentrated on a single chronic disease where the reporting of comorbid conditions was often a secondary variable or outcome measure and not an objective of the study. This review not only highlights the need for increased research into the study of multimorbidity or comorbidities of chronic diseases, but also the need for such studies to be of greater methodological rigour, given that almost a third of the studies included in this review scored only 50% using the critical appraisal tool. A similar disparity between the prevalence of multimorbidity in the general population of Canada and the number of research publications [13], was recently reported by Fortin and colleagues where the majority of studies focused on the epidemiology of multimorbidity or its effects on quality of life and physical functioning [14,22].

Our study has several limitations. The first is the risk of not finding all relevant Australian articles. We only used Medline with Ovid as the search engine and the search of other databases may have increased the likelihood of finding any additional papers. However, we felt that the use of all the key index chronic diseases as MeSH terms in addition to multimorbidity and comorbidity would have helped to ensure the identification all relevant articles. Furthermore, in accord with the number of chronic diseases and the diversity of articles included in this review, there was considerable disparity between studies in the methodological approach (the types of data used) and the classification of diseases and was reflected in the diversity of scores from the critical appraisal tool. Whilst self-report of medical conditions is an important tool in epidemiology research, its reported accuracy when compared with medical records, which are considered the 'gold-standard' of clinical data collection, appears to be disease specific and may be greatest for chronic disorders requiring ongoing management [58,59]. This was also evident in our study, where a general consensus of self-report with medical records was observed. The prevalence of comorbidities from hospital administrative data were generally lower when compared with other types of data, similar trends have previously been reported [48,60]. The classification of diseases was sometimes problematic particularly for CVD that incorporates a number of specific disease entities, such as IHD or CHF. These differences in data types and classification of disorders, in combination with a marked lack of studies, particularly for arthritis, asthma, cancer and mental health problems, made direct comparisons between studies sometimes difficult. Nevertheless, distinct patterns of comorbid conditions were evident, particularly for the older population. Arthritis, HT, CVD and diabetes were clearly the most prominent co-morbidities reported in this review. Whilst a strong relation between age and the prevalence of many chronic diseases exists, it does not negate from the importance and consequences of comorbidity.

Key issues for future study of multimorbidity that are not only pertinent to Australia, but world-wide, include the need to further define the populations of patients with multiple chronic diseases and their medication patterns. What is appropriate prescribing for patients with multimorbidity and what overall benefit may be achieved with complex regimens of medications? Reductions in mortality from heart disease in Australia have been achieved over the past couple of decades, with a combination of knowledge, prevention strategies and improvements in treatment options. The same can be achieved for patients with chronic disease and the ever growing population of patients with multimorbidity.

## Conclusion

Despite the high reported prevalence of patients with multiple morbidities, there are comparatively few Australian studies that focused on comorbidity associated with chronic disease. These studies do however demonstrate the high prevalence of comorbidity across national health priority areas, highlighting the need for integration and co-ordination of the national health priority areas. Clearly there is a need for more studies on multimorbidity to provide a strong evidence base on which to develop appropriate guidelines for the care and management of these patients.

## Competing interests

The authors declare that they have no competing interests.

## Authors' contributions

GEC conducted the literature search, summarized and critically appraised all eligible papers, conducted the synthesis of findings and the drafting of manuscript. AIV, ALG and EER participated in synthesis of findings and editing of manuscript.

## Pre-publication history

The pre-publication history for this paper can be accessed here:



## References

[B1] Australian Bureau of Statistics (2006). Population projections, Australia, 2004–2101.

[B2] Australian Bureau of Statistics (2006). National Health Survey: Summary of Results 2004–2005.

[B3] National Health Priority Action Council (NHPA) (2006). National chronic disease strategy.

[B4] Dowrick C (2006). The chronic disease strategy for Australia. Med J Aust.

[B5] Australian Institute of Health and Welfare (AIHW) (2006). Chronic diseases and associated risk factors in Australia, 2006.

[B6] World Health Organisation (2005). Preventing chronic disease: a vital investment.

[B7] Akker M van den, Buntinx F, Metsemakersa JFM, Roosb S, Knottnerus JA (1998). Multimorbidity in General Practice: Prevalence, Incidence, and Determinants of Co-Occurring Chronic and Recurrent Diseases. J Clin Epidemiol.

[B8] Wolff JL, Starfield B, Anderson G (2002). Prevalence, Expenditures, and Complications of Multiple Chronic Conditions in the Elderly. Arch Intern Med.

[B9] Fortin M, Bravo G, Hudon C, Vanasse A, Lapointe L (2005). Prevalence of Multimorbidity Among Adults Seen in Family Practice. Ann Fam Med.

[B10] Gijsen R, Hoeymans N, Schellevis FG, Ruwaard D, Satariano WA, Bos GA van den (2001). Causes and consequences of comorbidity: A review. J Clin Epidemiol.

[B11] Fortin M, Bravo G, Hudon C, Lapointe L, Dubois MF, Almirall J (2006). Psychological Distress and Multimorbidity in Primary Care. Ann Fam Med.

[B12] Fortin M, Bravo G, Hudon C, Lapointe L, Almirall J, Dubois MF, Vanasse A (2006). Relationship between multimorbidity and health-related quality of life of patients in primary care. Qual Life Res.

[B13] Fortin M, Lapointe L, Hudon C, Vanasse A (2005). Multimorbidity is common to family practice. Is it commonly researched?. Can Fam Physician.

[B14] Fortin M, Soubhi H, Hudon C, Bayliss EA, Akker M van den (2007). Multimorbidity's many challenges. BMJ.

[B15] Akker M van den, Buntinx F, Knottnerus JA (1996). Comorbidity or multimorbidity: what's in a name? A review of the literature. The European Journal of General Practice.

[B16] Akker M van den, Buntinx F, Roos S, Knottnerus JA (2001). Problems in determining occurrence rates of multimorbidity. J Clin Epidemiol.

[B17] Goldney R, Fisher L (2005). Use of prescribed medications in a South Australian community sample. Med J Aust.

[B18] Byles JE, Heinze R, Nair B, Parkinson L (2003). Medication use among older Australian veterans and war widows. Int Med J.

[B19] Burgess CL, Holman CD, Satti AG (2005). Adverse drug reactions in older Australians, 1981–2002. Med J Aust.

[B20] Elliott R (2006). Problems with medication use in the elderly: an Australian perspective. J Pharm Pract Res.

[B21] Zhang M, Holman CD, Preen DB, Brameld K (2007). Repeat adverse drug reactions causing hospitalization in older Australians: a population-based longitudinal study 1980–2003. BJ Clin Pharm.

[B22] Tinetti ME, Bogardus ST, Agostini JV (2004). Potential Pitfalls of Disease-Specific Guidelines for Patients with Multiple Conditions. N Engl J Med.

[B23] Boyd CM, Darer J, Boult C, Fried LP, Boult L, Wu AW (2005). Clinical Practice Guidelines and Quality of Care for Older Patients With Multiple Comorbid Diseases: Implications for Pay for Performance. JAMA.

[B24] van Weel C, Schellevis FG (2006). Comorbidity and guidelines: conflicting interests. Lancet.

[B25] Van Spall HG, Toren A, Kiss A, Fowler RA (2007). Eligibility Criteria of Randomized Controlled Trials Published in High-Impact General Medical Journals: A Systematic Sampling Review. JAMA.

[B26] Australian Institute of Health and Welfare: National Health Priority Areas. http://www.aihw.gov.au/nhpa/.

[B27] Begg S, Vos T, Barker B, Stevenson C, Stanley L, Lopez AD (2007). The burden of disease and injury in Australia 2003.

[B28] Loney PL, Chambers LW, Bennett KJ, Roberts JG, Stratford PW (1998). Critical appraisal of the health research literature: prevalence or incidence of a health problem. Chronic Dis Canada.

[B29] Hill CL, Gill T, Taylor AW, Daly D, Dal Grande E, Adams RJ (2007). Psychological factors and quality of life in arthritis: a population-based study. Clinical Rheumatology.

[B30] Adams RJ, Wilson DH, Taylor AW, Daly A, d'Espaignet ET, Dal Grande E, Ruffin RE (2006). Coexistent Chronic Conditions and Asthma Quality of Life: A Population-Based Study. Chest.

[B31] Goldney RD, Ruffin R, Fisher LJ, Wilson DH (2003). Asthma symptoms associated with depression and lower quality of life: a population survey. Med J Aust.

[B32] Goldney RD, Phillips PJ, Fisher LJ, Wilson DH (2004). Diabetes, Depression, and Quality of Life: A population study. Diabetes Care.

[B33] Kemp TM, Barr ELM, Zimmet PZ, Cameron AJ, Welborn TA, Colagiuri S, Phillips P, Shaw JE, on behalf of the AusDiab Steering Committee (2005). Glucose, Lipid, and Blood Pressure Control in Australian Adults With Type 2 Diabetes: The 1999–2000 AusDiab. Diabetes Care.

[B34] Mitchell P, Smith W, Wang JJ, Cumming RG, Leeder SR (1998). Diabetes in an older Australian population. Diab Res Clin Prac.

[B35] Waghorn G, Chant D, Lloyd C (2006). Labor force activity among Australians with musculoskeletal disorders comorbid with depression and anxiety disorders. J Occup Rehabil.

[B36] Pilotto LS, Smith BJ, Heard AR, McElroy HJ, Weekley J, Bennett P (2004). Trial of nurse-run asthma clinics based in general practice versus usual medical care. Respirology.

[B37] Parker G, Heruc G, Hilton T, Olley A, Brotchie H, Hadzi-Pavlovic D, Owen C, Friend C, Walsh WF (2006). Explicating links between acute coronary syndrome and depression: study design and methods. Aust N Z J Psychiatry.

[B38] Bruce DG, Davis WA, Starkstein SE, Davis TM (2005). A prospective study of depression and mortality in patients with type 2 diabetes: the Fremantle Diabetes Study. Diabetologia.

[B39] Burvill P, Johnson G, Jamrozik K, Anderson C, Stewart-Wynne E (1997). Risk factors for post-stroke depression. Int J Ger Psych.

[B40] Clarke P, Kelman C, Colagiuri S (2006). Factors influencing the cost of hospital care for people with diabetes in Australia. J Diabetes Complications.

[B41] Stewart S, Blue L, Capewell S, Horowitz JD, McMurray JJ (2001). Poles apart, but are they the same? A comparative study of Australian and Scottish patients with chronic heart failure. Eur J Heart Failure.

[B42] O'Halloran J, Britt H, Valenti L, Harrison C, Pan Y, Knox S (2003). Older Patients Attending General Practice in Australia 2000–02. AIHW Cat. No. GEP 12.

[B43] Thomas MC, Nestel PJ (2007). Management of dyslipidaemia in patients with type 2 diabetes in Australian primary care. Med J Aust.

[B44] Krum H, Meehan A, Varigos J, Loane PR, Billah B (2006). Does the presence of heart failure alter prescribing of drug therapy after myocardial infarction? A multicentre study. Med J Aust.

[B45] Simons LA, Simons J, Friedlander Y, McCallum J (2002). Risk factors for acute myocardial infarction in the elderly (the Dubbo study). Am J Cardiol.

[B46] Krum H, Tonkin AM, Currie R, Djundjek R, Johnston CI (2001). Chronic heart failure in Australian general practice. The Cardiac Awareness Survey and Evaluation (CASE) Study. Med J Aust.

[B47] Sturm JW, Donnan GA, Dewey HM, Macdonell RA, Gilligan AK, Thrift AG (2004). Determinants of Handicap After Stroke: The North East Melbourne Stroke Incidence Study (NEMESIS). Stroke.

[B48] Powell H, Lim L, Heller R (2001). Accuracy of administrative data to assess comorbidity in patients with heart disease: an Australian perspective. J Clin Epidemiol.

[B49] Hunt C, Slade T, Andrews G (2004). Generalized Anxiety Disorder and Major Depressive Disorder comorbidity in the National Survey of Mental Health and Well-Being. Depression and Anxiety.

[B50] Wilson I, Duszynski K, Mant A (2003). A 5-year follow-up of general practice patients experiencing depression. Fam Pract.

[B51] Australian Bureau of Statistics (1997). National Survey of Mental Health and Wellbeing of Adults.

[B52] Valery PC, Coory M, Stirling J, Green AC (2006). Cancer diagnosis, treatment, and survival in Indigenous and non-Indigenous Australians: a matched cohort study. Lancet.

[B53] Huxley R, Barzi F, Woodward M (2006). Excess risk of fatal coronary heart disease associated with diabetes in men and women: meta-analysis of 37 prospective cohort studies. BMJ.

[B54] Starfield B, Lemke KW, Bernhardt T, Foldes SS, Forrest CB, Weiner JP (2003). Comorbidity: Implications for the Importance of Primary Care in 'Case' Management. Ann Fam Med.

[B55] Bodenheimer T, Lorig K, Holman H, Grumbach K (2002). Patient Self-management of Chronic Disease in Primary Care. JAMA.

[B56] Coleman MT, Newton KS (2005). Supporting self-management in patients with chronic illness. Am Fam Physician.

[B57] Kwoh CK, Ibrahim SA (2001). Rheumatology patient and physician concordance with respect to important health and symptom status outcomes. Arthritis Care & Research.

[B58] Merkin SS, Cavanaugh K, Longenecker JC, Fink NE, Levey AS, Powe NR (2007). Agreement of self-reported comorbid conditions with medical and physician reports varied by disease among end-stage renal disease patients. J Clin Epidemiol.

[B59] Okura Y, Urban LH, Mahoney DW, Jacobsen SJ, Rodeheffer RJ (2004). Agreement between self-report questionnaires and medical record data was substantial for diabetes, hypertension, myocardial infarction and stroke but not for heart failure. J Clin Epidemiol.

[B60] Preen DB, Holman CDJ, Lawrence DM, Baynham NJ, Semmens JB (2004). Hospital chart review provided more accurate comorbidity information than data from a general practitioner survey or an administrative database. J Clin Epidemiol.

